# Mitochondrial fragmentation and ROS signaling in wound response and repair

**DOI:** 10.1186/s13619-022-00141-8

**Published:** 2022-12-01

**Authors:** Shiqi Xu, Shiyao Li, Mikael Bjorklund, Suhong Xu

**Affiliations:** 1 Zhejiang University-University of Edinburgh Institute and International Biomedicine-X Research Center of the Second Affiliated Hospital, Zhejiang University School of Medicine, 718 East Haizhou Rd., Haining, 314400 Zhejiang China; 2 Center for Stem Cell and Regenerative Medicine and Department of Burn and wound repair of the Second Affiliated Hospital, Zhejiang University School of Medicine, Hangzhou, 310058 Zhejiang China

**Keywords:** Mitochondrial dynamic, Reactive oxygen species, Plasma membrane, Membrane repair, Mitochondrial fragmentation, MIRO-1, MFN-1/2, FZO-1

## Abstract

Mitochondria are organelles that serve numerous critical cellular functions, including energy production, Ca^2+^ homeostasis, redox signaling, and metabolism. These functions are intimately linked to mitochondrial morphology, which is highly dynamic and capable of rapid and transient changes to alter cellular functions in response to environmental cues and cellular demands. Mitochondrial morphology and activity are critical for various physiological processes, including wound healing. In mammals, wound healing is a complex process that requires coordinated function of multiple cell types and progresses in partially overlapping but distinct stages: hemostasis and inflammation, cell proliferation and migration, and tissue remodeling. The repair process at the single-cell level forms the basis for wound healing and regeneration in tissues. Recent findings reveal that mitochondria fulfill the intensive energy demand for wound repair and aid wound closure by cytoskeleton remodeling via morphological changes and mitochondrial reactive oxygen species (mtROS) signaling. In this review, we will mainly elucidate how wounding induces changes in mitochondrial morphology and activity and how these changes, in turn, contribute to cellular wound response and repair.

## Background

**Fig. 1:**
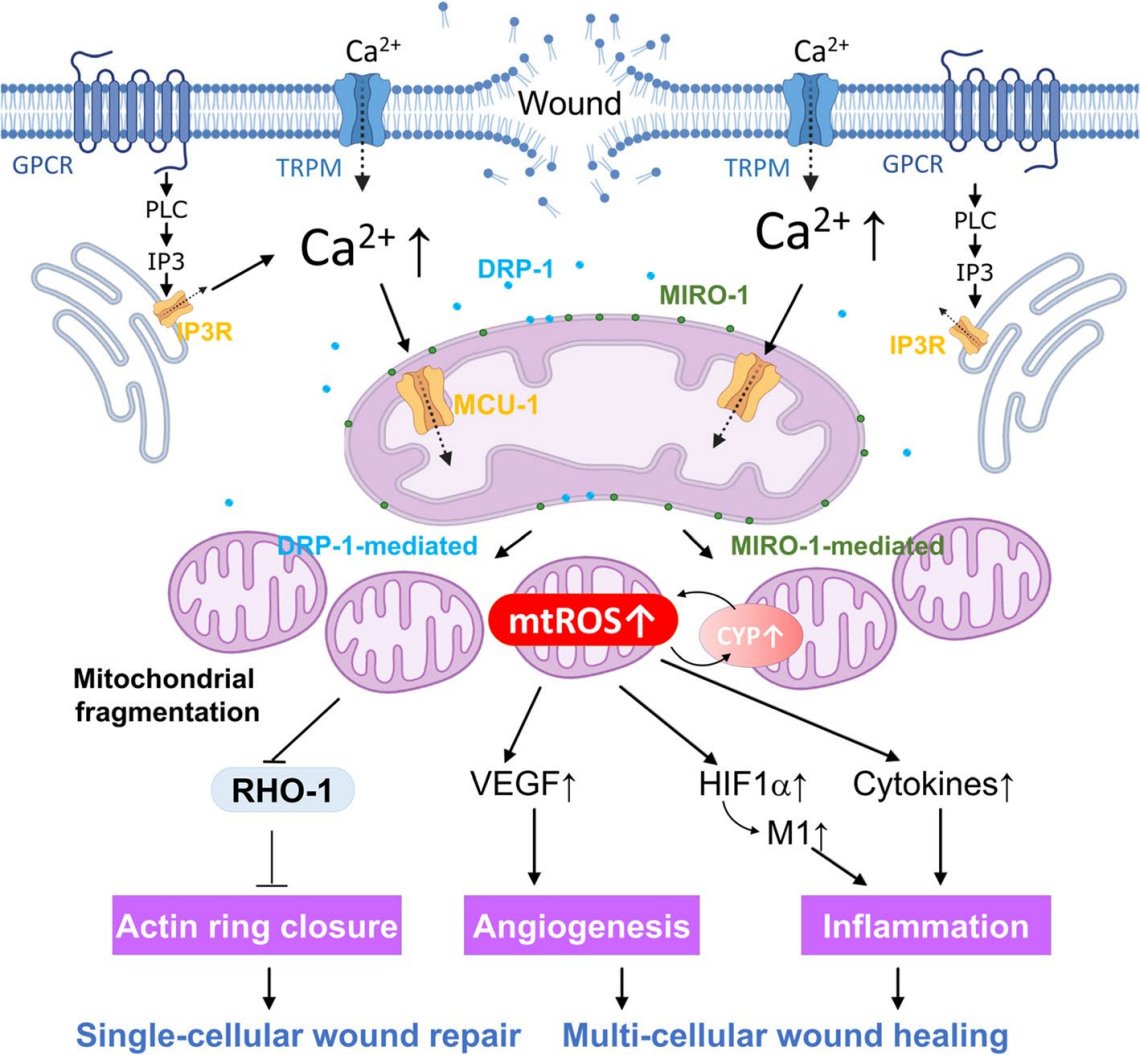
A model of mitochondrial fragmentation and ROS signaling in regulating wound repair. Mitochondria play multiple roles in both single-cellular wound closure and multicellular wound healing. Tissue wound induces an acute elevation of cytosolic Ca^2+^ through an external influx or internal release, leading to mitochondrial fragmentation dependent on DRP-1 or MIRO-1. As a further response to calcium, the fragmented mitochondria increase mtROS production, promoting actin ring closure in a single cell system. mtROS may also facilitate multicellular wound healing by being involved in angiogenesis and inflammation processes

Mitochondria are semi-autonomous and double-membrane-bound organelles in eukaryotic cells with a well-known role in adenosine triphosphate (ATP) production via oxidative phosphorylation (OXPHOS). The ATP production machinery requires five protein complexes (Complex I-V) embedded in the inner mitochondrial membrane (IMM) and two mobile electron carriers (Vercellino and Sazanov 2022). In addition to this bioenergetic role, mitochondria are one the predominant source of reactive oxygen species (ROS), byproducts of OXPHOS mainly attributable to Complex I and III (Murphy 2008). Resultant mitochondrial ROS (mtROS) enters the cytoplasm to promote redox signaling, mediating various biological responses, including cell proliferation, differentiation, and migration (Fig. 1) (Holmström and Finkel 2014).

Mitochondria are also remarkably dynamic, with morphology ranging from individual fragmented punctate spheres to highly interconnected reticular networks. These morphologies are controlled by antagonizing fusion and fission events (Chan 2012). The machinery underlying these events is well-conserved, with guanosine triphosphatases (GTPases) in the dynamin family as core regulators. Mitochondrial fission is accomplished by dynamin-related protein 1 (DRP1) (Vepa et al. 1999). Upon activation, this large cytosolic GTPase is anchored to the outer mitochondrial membrane (OMM) by its adaptor proteins, such as mitochondrial dynamics protein 49 (MiD49), fusion 1 protein (FIS1), and oligomerizes into a ring-like structure that constricts to sever the parent mitochondrion into two daughter mitochondria (Loson et al. 2013). Apart from the canonical DRP1-dependent pathway, various studies reveal that a rapid mitochondrial shape transition can be achieved by other molecular machinery, including actin-dependent mitochondrial fission, endoplasmic reticulum (ER), FUNDC1, and myosin-regulated mitochondrial division (Chen et al. 2016; Korobova et al. 2014, 2013; Loson et al. 2013; Wu et al. 2016). Mitochondrial Rho GTPase MIRO-1 has also been found to regulate mitochondrial morphology in multiple organisms(Ding et al. 2016, R.L. et al. 2004, Xu et al. 2016). MIRO-1 regulates mitochondrial dynamics by the cytosolic Ca^2+^ signal (Ding et al. 2016; Nemani et al. 2018). As for mitochondrial fusion, it entails the merging of both OMM and IMM. The former is executed by mitofusin 1 and 2 (MFN 1 and 2), while the latter is mediated by optic atrophy protein 1 (OPA1) (Youle and Bliek 2012). These changes in mitochondrial morphology are intimately linked to mitochondrial activity, which influences the production of both ATP and mtROS, allowing mitochondria to respond to environmental stimuli and adapt to cellular demands (Sabouny and Shutt 2020).

Wound healing following injuries is essential for the survival of all multicellular organisms (Gurtner et al. 2008). In mammals, it is a complex process that involves coordinated function of multiple cell types and proceeds in overlapping but distinct stages: hemostasis and inflammation, cell proliferation and migration, and tissue remodeling (Gurtner et al. 2008, Madan et al. 2022). Hemostasis occurs immediately after an injury when coagulation cascade and blood vessel resealing are activated to prevent excessive blood loss. Simultaneously, inflammatory cells, including neutrophils and macrophages, are recruited to the wound site to defend against pathogen invasion (Willenborg et al. 2021). Eventually, the epithelial cell proliferates, differentiates, and migrates to re-epithelialize the wounded site. Single-cell wound repair progresses essentially in a similar manner as tissue damage repair (Sonnemann and Bement 2011). Knowing how a single cell repairs and regenerates itself helps to understand mechanistically the cell biology involved in tissue damage (Tang and Marshall 2017). Accumulating evidence suggests that mitochondria play important roles in these stages through changes in their morphology and activity (Horn and Jaiswal 2020; Schiffmann et al. 2020). In this review, we mainly discuss how wounding induces these mitochondrial morphological changes and how these changes, in turn, contribute to wound response and repair in a cellular system.

## Wound-induced elevation in Ca^2+^ leads to changes in mitochondrial morphology

Elevation of cytosolic Ca^2+^ in cells around the wound margin is conserved across species (Wood 2012). Ca^2+^ is the earliest transcription-independent signal to injury (activated in milliseconds) and can activate downstream targets to achieve multiple functions in wound repair, including inflammation and actomyosin-regulated wound repair (Cordeiro and Jacinto 2013). However, the mechanisms underlying the elevation of Ca^2+^ can be quite diverse. For example, our laboratory uses *C. elegans* syncytium epidermal cell hyp7 as a genetically tractable model to study wound repair and repair (Ma et al. 2021). In this cell, Ca^2+^ enters the extracellular space directly through transient receptor potential (TRP) channels family on the plasma membrane, such as GTL-2 (Antunes et al. 2013; Razzell et al. 2013, Xu and Chisholm 2011) (Fig. 1). In others, Ca^2+^ is released from the internal stores, such as the endoplasmic reticulum, via inositol-1,4,5-triphosphate (IP_3_) receptor-activated by G-protein-coupled receptor (GPCR) signaling. Regardless of the initial source, intracellular Ca^2+^ burst and its wave-like propagation enable rapid wound detection (Wood 2012).

Mitochondria undergo rapid and reversible fragmentation after wounding and this depends on cytosolic Ca^2+^ increase (Fu et al. 2020, Horn et al. 2020, Ponte et al. 2020). This response is also spatially restricted, with injury-proximal fragmented mitochondria isolated from injury-distal interconnected mitochondria. With different cell types, the wounding-induced mitochondrial shape change is dependent on different molecular machinery (Horn and Jaiswal 2020). This may be due to inherent differences between cell types, which affect the expression of specific regulators. Wound-induced fragmentation of mitochondria is commonly driven by DRP-1 (Ponte et al. 2020). Ablation of DRP-1 and its adaptor MiD49 in mammalian cells results in a hyper-elongated mitochondrial network that fails to fragment upon focal membrane injury, leading to compromised plasma membrane repair (Horn et al. 2020) (Fig. 1). In *Drosophila* embryos with different *drp-1* mutants, two types of epidermal wound closure phenotypes are observed, a mild one with a significantly slower closure rate and a strong one with an expanded wound area (Ponte et al. 2020). However, how DRP1 senses the damage and induces mitochondrial division remains unknown. Wound-induced mitochondrial fragmentation is also observed in *C. elegans* following epidermal injury. The local mitochondrial network at the site of injury becomes first fragmented, which is followed by fragmentation of surrounding mitochondria (50–70 μm from the wound site) within minutes (Fu et al. 2020). This fragmentation, however, is independent of DRP1, as both knockdown of *drp-1* and *drp-1* deletion mutant show normal mitochondrial fragmentation after wounding (Fu et al. 2020). This study also finds that spreading mitochondrial fragmentation requires MIRO-1 (Fu et al. 2020) (Fig. 1), an adaptor on OMM that is important for mitochondrial transport (Cai and Sheng 2009). MIRO-1 functions as a Ca^2+^ sensor on the OMM and can directly sense the wounding-induced elevation of Ca^2+^ that triggers the rapid mitochondrial fragmentation.

MIRO-1 is involved in mitochondrial transport along the microtubule and directly interacts with Trak-1, a kinesin protein involved in trafficking (Debattisti et al. 2017). However, knockdown of *trak-1* in *C. elegans* doesn’t affect wounding-induced mitochondrial fragmentation. Moreover, neither does the inhibition of microtubule dynamic, suggesting wound-induced mitochondrial fragmentation is not microtubule dependent (Fu et al. 2020). The underlying molecular mechanism of MIRO-1 in controlling mitochondrial fragmentation remains to be clarified. It is possible that other mitochondrial fission machinery could interact with MIRO-1 to regulate mitochondrial fission, or MIRO-1 itself could function directly on mitochondrial fission. It is known that loss of mitochondrial membrane potential results in mitochondrial fragmentation (Zorova et al. 2018); whether MIRO-1 is involved in regulating mitochondrial membrane potential remains unknown. In *Drosophila* embryonic wounding, DRP-1 regulates injury-induced mitochondrial division, consistent with findings in mammalian cells (Horn et al. 2020, Ponte et al. 2020). While both DRP-1 and MIRO-1 regulate mitochondrial dynamics, it remains unclear if DRP-1 and MIRO-1 function redundantly in response to wounding or whether there is any genetic or physical interaction between MIRO-1 and DRP-1. MIRO-1 is also necessary for the wound-induced dendrite regeneration of PVD sensory neurons, which respond to high threshold mechanical stimuli in *C. elegans* (Zhao et al. 2021). Altogether, these findings demonstrate that Ca^2+^-dependent mitochondrial fragmentation is a common response to injury and allows efficient repair of wounds.

## Mitochondrial fragmentation activates the local production of ROS

Elevation in cytosolic Ca^2+^ triggers rapid mitochondrial Ca^2+^ uptake via mitochondrial calcium uniporter (MCU), which contributes to wound healing by transiently enhancing mitochondrial reactive oxygen species (mtROS) production (Xu and Chisholm 2014). This response was first visualized in *C. elegans* epidermis following injury by mitochondrial superoxide sensor mito::cpYFP, and is ablated in MCU-1 mutants (Xu and Chisholm 2014). Although the precise mechanism underlying this enhanced mtROS production remains enigmatic, the opening of mitochondrial permeability transition pore triggered by mitochondrial Ca^2+^ uptake, and wounding-induced mitochondrial fragmentation, are proposed to be involved (Ma et al. 2021). Similarly, a drastic increase in mtROS is observed in delaminating cells during *Drosophila* dorsal closure, a late-embryogenesis stage resembling wound healing (Muliyil and Narasimha 2014) and embryonic wound healing (Hunter et al. 2018). Mitochondrial Ca^2+^ uptake stimulates OXPHOS to enhance the production of ATP and its byproduct mtROS (Liu and O'Rourke 2008). This ATP synthesis is dispensable for skeletal muscle repair, where mtROS exhibits a dose-dependent beneficial impact on wound healing (Le Moal et al. 2017). These findings suggest that wound-induced mtROS burst plays a conserved role in cellular wound repair. While this highlights the benefit of acute mitochondrial ROS signaling for wound repair, the question remains of how mitochondrial ROS signaling is localized to the injury site.

## mtROS promote wound closure in a single cell

ROS mainly arises from mitochondria, peroxisomes, and the endoplasmic reticulum but can also be generated in the cytoplasm. The best characterized intracellular sources of ROS are mitochondria and NADPH oxidases on the plasma membrane (Holmström and Finkel 2014). Homeostatic ROS is required for both cellular wound closure and multicellular wound healing processes, including hemostasis, inflammation, and tissue remodeling (Dunnill et al. 2017; Rives et al. 2020). Epidermal wound healing in *C. elegans* requires the formation of an actin ring surrounding the wound site via rapid actin polymerization, which is mediated by CDC42 and negatively regulated by RHO-1 (Ma et al. 2021, Xu et al. 2021, Xu and Chisholm 2011). Ca^2+^-triggered mtROS increase at the *C. elegans* epidermal wound site can promote wound repair. Inhibiting mtROS by antioxidants blocks actin-based wound closure, while the elevation of mtROS by genetic mutation enhances wound healing (Xu and Chisholm 2014). By targeting a redox-sensitive motif, mtROS can inhibit RHO-1 activity, which negatively regulates actin ring closure (Xu and Chisholm 2014) (Fig. 1). A similar mechanism is observed in injured skeletal muscle cells (Horn et al. 2017). Calcium triggers mtROS production, facilitating wound closure through activating RhoA to promote F-actin accumulation. Later, it was found that upregulation of mtROS is required for the enhanced wound closure in the *fzo-1* (human MFN1/2 homology) mutant *C. elegans* where the mitochondria are constitutively fragmented (Fu et al. 2020).

Increased mtROS production and the upregulation of several oxidative signals genes, including cytochrome P450 (cyp) family genes, are detected in *fzo-1* mutants compared to WT animals after wounding (Fu et al. 2020). CYPs can generate ROS, while ROS can reversely upregulate CYPs expression (Zhao et al. 2017). RNAi knockdown of *cyp* genes can lead to decreased mtROS levels and inhibit actin ring closure, while their overexpression accelerates actin ring closure in both *fzo-1* mutants and WT animals. Similarly, antioxidants treatments in *fzo-1* mutants decrease ROS levels and further delay wound closure, while treatment with mitochondrial Complex I inhibitor rotenone, which induces ROS production, can revert these effects (Fu et al. 2020). During *Drosophila* dorsal closure, mtROS can trigger cell delamination (Muliyil and Narasimha 2014). It was found that mtROS can drive mitochondrial fragmentation through Drp1 and caspase activation and that mtROS influences cytoskeleton rearrangement through the Rho Effector ROCK and pMLC pathway (Muliyil and Narasimha 2014). In laser-wounded *Drosophila* embryonic epidermis, mitochondrial fission defects can also lead to reduced mtROS levels and delayed wound closure (Ponte et al. 2020). These results suggest that an elevated mtROS level is necessary and sufficient for enhanced wound closure *in C. elegans* epidermis.

## Mitochondrial ROS facilitates wound healing in multicellular organisms

mtROS is also required for damaged tissue homeostasis, mediating vasoconstriction and thrombus formation. Antioxidant treatment can inhibit the vasoconstriction upon cold exposure in mice, and ROS is involved in vasoconstriction via the ROS/RhoA/ROCK1 and ROS/PKC/ET-1 pathways in vascular smooth muscle cells and endothelial cells (Zhang et al. 2021). Vasculature-restricted respiratory deficiency through *cox10* ablation causes embryonic lethality in mice, while in adult mice, *cox10* deficiency shows defective angiogenic capacity near wounds (Schiffmann et al. 2020). Cell-permeable mitochondrial ubiquinol–cytochrome c reductase binding protein (UQCRB) can increase mtROS production, which induces vascular endothelial growth factor (VEGF) expression and enhances angiogenesis and cutaneous wound healing in mice (Chang et al. 2015).

mtROS is responsible for both the production of proinflammatory cytokines after lipopolysaccharide (LPS) stimulation in normal cells and hyperproduction of cytokines in cells from patients with tumor necrosis factor receptor-associated periodic syndrome (TRAPS), an autoinflammatory disease (Bulua et al. 2011). mtROS are upregulated through coupling TLR1/2/4 signaling to mitochondrial Complex I via TNF receptor-associated factor 6 (TRAF6) and evolutionarily conserved signaling intermediate in Toll pathway (ECSIT). Deleting TRAF6 and ECSIT or overexpressing antioxidant enzymes in mitochondria results in decreased mtROS and defective bacterial-killing (West et al. 2011). In the early stage of skin wound healing in mice, macrophages show hallmarks of a dysfunctional TCA cycle as well as increased mtROS production. Increased mtROS affects HIF1α stabilization, converting macrophages into M1 subtype, which promotes vascularization and inflammation during wound healing (Willenborg et al. 2021). Thus, mtROS signaling is essential for wound healing at both single-cell and multi-cell levels. However, it is important to realize that excessive and uncontrolled ROS production can damage tissue and lead to non-healing wounds in diseases such as diabetes (Deng et al. 2021). Consistently, applying oxygen-releasing and ROS–scavenging hydrogel to diabetic wounds in mice may promote wound healing by augmenting angiogenesis, re-epithelialization, and inhibiting inflammation (Guan et al. 2021).

## Conclusions

Accumulating evidence suggests that mitochondrial dynamics and function respond to wounding and will lead to changes in the downstream signals that participate in the wound healing process. As one of the primary sources of ROS, mitochondria are an important player in generating these signals. ROS can directly affect protein function through posttranscriptional modification or indirectly by regulating protein expression. While mitochondria and ROS are promising targets to promote wound healing, it is essential to consider ROS's biphasic and dose-dependent roles and the different functions of mitochondria and mitochondrial ROS in various cell types. It is also important to identify more details and underlying mechanisms to achieve higher efficiency and reduction in side effects. For example, identifying the threshold level of detrimental mtROS should be helpful for personalized medicine. As wound healing is a complex process involving various cell types, it is important to explore the functions of mitochondria in the interaction between cells in addition to focusing on just one cell type. In addition, the stage-specific roles of mtROS in wound healing are still poorly defined. Furthermore, the endogenous enzymatic and non-enzymatic factors involved in antioxidant defense systems that guard against mtROS should also be considered in wound healing. Future development and application of real-time sensors to detect how mitochondria sense the wounding in model organisms such as C. elegans will further help the study of the highly dynamic wound healing process.

## Data Availability

Not applicable.

## Abbreviations

ATPL: Adenosine triphosphate
CDC42: Cell division cycle 42
COX10: Cytochrome C oxidase assembly factor heme A:farnesyltransferase COX10
CYP: Cytochrome P450
DRP1: Dynamin-related protein 1
ECSIT: Evolutionarily conserved signaling intermediate in Toll pathway
ER: Endoplasmic reticulum
ET-1: Endothelin-1
FIS1: Fusion 1 protein
FUNDC1: FUN14 domain containing 1
GPCR: G-protein-coupled receptor
GTL-2: Gon-two like
GTPases: Guanosine triphosphatases
IMM: Inner mitochondrial membrane
IP3: Inositol-1,4,5-triphosphate
LPS: Lipopolysaccharide
MCU: Mitochondrial calcium uniporter
MFN: Mitofusin
MiD49: Mitochondrial dynamics protein 49
mtROS: Mitochondrial reactive oxygen species
NADPH: Nicotinamide adenine dinucleotide phosphate oxidase
OMM: Outer mitochondrial membrane
OPA1: Optic atrophy protein 1
OXPHOS: Oxidative phosphorylation
PKC: Protein kinase C
pMLC: Phosphorylated myosin light chain
RHO-1: RhoA ortholog in C.elegans
ROCK: Rho associated coiled-coil containing protein kinase 1
ROS: Reactive oxygen species
TLR: Toll-like receptor
TRAPS: Tumor necrosis factor receptor-associated periodic syndrome
TRP: Transient receptor potential
UQCRB: Ubiquinol–cytochrome c reductase binding protein
VEGF: Vascular endothelial growth factor
YFP: Yellow fluorescent protein
